# Quantification and Kinetic Analysis of Grb2-EGFR Interaction on Micro-Patterned Surfaces for the Characterization of EGFR-Modulating Substances

**DOI:** 10.1371/journal.pone.0092151

**Published:** 2014-03-21

**Authors:** Peter Lanzerstorfer, Daniela Borgmann, Gerhard Schütz, Stephan M. Winkler, Otmar Höglinger, Julian Weghuber

**Affiliations:** 1 University of Applied Sciences Upper Austria, Wels, Austria; 2 University of Applied Sciences Upper Austria, Hagenberg, Austria; 3 Institute of Applied Physics, Vienna University of Technology, Vienna, Austria; Hungarian Academy of Sciences, Hungary

## Abstract

The identification of the epidermal growth factor receptor (EGFR) as an oncogene has led to the development of several anticancer therapeutics directed against this receptor tyrosine kinase. However, drug resistance and low efficacy remain a severe challenge, and have led to a demand for novel systems for an efficient identification and characterization of new substances. Here we report on a technique which combines micro-patterned surfaces and total internal reflection fluorescence (TIRF) microscopy (μ-patterning assay) for the quantitative analysis of EGFR activity. It does not simply measure the phosphorylation of the receptor, but instead quantifies the interaction of the key signal transmitting protein Grb2 (growth factor receptor-bound protein 2) with the EGFR in a live cell context. It was possible to demonstrate an EGF dependent recruitment of Grb2 to the EGFR, which was significantly inhibited in the presence of clinically tested EGFR inhibitors, including small tyrosine kinase inhibitors and monoclonal antibodies targeting the EGF binding site. Importantly, in addition to its potential use as a screening tool, our experimental setup offers the possibility to provide insight into the molecular mechanisms of bait-prey interaction. Recruitment of the EGFR together with Grb2 to clathrin coated pits (CCPs) was found to be a key feature in our assay. Application of bleaching experiments enabled calculation of the Grb2 exchange rate, which significantly changed upon stimulation or the presence of EGFR activity inhibiting drugs.

## Introduction

Receptor tyrosine kinases (RTKs) are a subclass of signaling receptors anchored at the cell surface and have intrinsic tyrosine kinase activity triggering signal transduction in response to ligand binding. RTKs are generally activated through ligand-induced oligomerization, typically dimerization, which leads to autophosphorylation of tyrosine residues in the kinase activation loop or the juxtamembrane region [1]. These phosphotyrosine residues are important docking sites for a plethora of intracellular downstream signaling molecules and are typically bound by Src homology-2 (SH2) or phosphotyrosine-binding (PTB) domains [2].

The epidermal growth factor receptor (EGFR) is a member of the ErbB family of receptors, a subclass of RTKs, and is expressed in all epidermal cells as well as stromal, glia and smooth muscle cells [3]. EGFR signaling is one of the most important pathways that regulate growth, survival, proliferation and differentiation in mammalian cells [4]. Thus, EGFR signaling is also critical for the development of many types of cancer. Mutations that lead to EGFR overexpression or overactivity have been associated with a number of cancers, including lung cancer, anal cancers and the glioblastoma multiforme [5], [6]. Mutations involving the EGFR may lead to its constant activation, which results in uncontrolled cell division. Consequently, mutations of the EGFR have been identified in several types of cancer and it is the target of an expanding class of anticancer therapies [7]–[9].

The identification of EGFR as an oncogene has led to the development of anticancer therapeutics directed against the EGFR including AG1478, Gefitinib, Erlotinib, Lapatinib, Canertinib (small molecule kinase inhibitors) and Cetuximab (monoclonal antibody inhibitor) [10]–[12]. Resistance to these drugs has emerged as a major clinical problem limiting the efficacy of currently used inhibitors and their use in cancer patients [13]. Thus, there is a demand for the optimization of existing, but also for the design of novel screening approaches to develop new inhibitors of RTKs.

Current approaches are mainly based on the screening of purified kinase domains against large chemical libraries. These *in-vitro* techniques suffer from several limitations, including the missing activity of many substances in a live cell context. Immunoblots and microarrays are also frequently used to quantify EGFR activation. Recently, an optimization of these approaches that allows for higher throughput has been reported [14]. However, necessary cell lysis and protein extraction steps exclude measurements in living cells. Phosphorylated EGFR can also be measured by a microarray method utilizing fluorescence lifetime imaging [15]. A drawback of this method, however, lies in the requirement for fluorescently tagged EGF, which precludes the investigation of endogenous EGFR. Furthermore, it is also not compatible with live-cell measurements. Immuno-histochemical methods in the microwell plate format [16], [17] also do not allow for the measurement of EGFR activity in live cells. A novel strategy has recently been reported by Antczak *et al*., who used a GFP labeled SHC homology 2 domain-based biosensor that induces green granules upon EGF stimulation in order to quantify endogenous EGFR [18]. In any case, there is a considerable necessity to develop new assays that facilitate the identification of novel EGFR modulators in a live cell context at sufficient throughput rates.

For the present study we used micro-patterned (μ-patterned) surfaces in combination with fluorescent microscopy to quantify the effects of EGFR activity modulating substances on downstream signaling events in living cells. We have recently introduced this assay to detect and quantify protein-protein interactions in a live cell context and address several different biological questions [19]–[25]. Here the μ-patterning technique was utilized to enrich endogenous bait EGFR in HeLa cells into microscopic domains, and monitor the co-recruitment of the fluorescent prey Grb2. This cytosolic protein is best known for its ability to link the EGFR tyrosine kinase to the activation of Ras and its downstream kinases, ERK1/2 [26]. Using our assay, we were able to confirm and quantify the recruitment of Grb2 to the EGFR in a phosphorylation dependent manner. Pretreatment with pharmacologically active ingredients that are applied for the treatment of human cancers significantly reduced the inducibility of the signaling system. Based on these results, we could set up a dose-response relationship in a live cell context. Our results clearly demonstrate the superior applicability of the μ-patterning assay to analyze the binding properties of medically relevant membrane receptors, and to identify and characterize substances which modulate the receptor’s activity. However, in addition to its usability as a potential screening tool, the present study provides insight into the EGFR-Grb2 binding kinetics. Our experimental setup enabled the quantification of the EGFR-Grb2 interaction strength (fluorescent contrast) as well as the residence time (exchange-rate) of Grb2 at the EGFR. Using photobleaching experiments we calculated the Grb2 exchange rate in individual clathrin coated pits (CCPs) and determined the differences under stimulated and non-stimulated conditions in living HeLa cells.

## Materials and Methods

### DNA constructs and reagent

The pEGFP-N1-EGFR plasmid was kindly provided by M. Matsuda (Kyoto University, Japan), the pYFP-N1-Grb2 by L.E. Samelson, (NIH, Bethesda) and the pEGFP-C1-CLCA1 construct was a kind gift from Eileen M. Lafer (UTHSC San Antonio, USA). The monoclonal antibody against the EGFR (clone EGFR1) was purchased from Antibodies online (Hereford, Germany). The EGFR inhibitors Gefitinib, Erlotinib, Canertinib and Lapatinib were from Synkinase (Shanghai, China). AG1478, EGF, DMSO and Transferrin-Alexa 647 were purchased from Sigma-Aldrich (Schnellendorf, Germany). Anti-EGFR monoclonal antibody Cetuximab (Erbitux) was a kind gift from Merck Serono (Vienna, Austria). All EGFR inhibitors (except for Cetuximab; PBS) were diluted in DMSO. DiD oil was purchased from Invitrogen (Carlsbad, California).

### Cell culture and transfection

RPMI, fetal bovine serum (FBS), antibiotics and Geneticin (G418 sulfate) were purchased from PAA Laboratories GmbH (Pasching, Austria). Human HeLa cells from the American Type Culture Collection were used. An electroporation unit (Nucleofector) and electroporation cuvettes were from Lonza (Basel, Switzerland). HeLa cells were cultured in RPMI medium supplemented with 10 % FBS and penicillin/streptomycin and grown at 37 °C in a humidified incubator (≥ 95 %) with 5 % CO_2_. For the generation of stable clones, cells were transfected with 1–5 μg DNA at 50–70 % confluence using the Nucleofector device. Cells were plated into 60 mm culture dishes and grown for 48 h. The medium was removed and replaced by medium supplemented with 400 μg/ml G418. This medium was changed every 3 days and 15–20 days later individual neomycin-resistant colonies were selected for propagation and analysis.

### μ-contact printing

μ-contact printing was performed as previously reported [21] except for a few modifications. In short, a μ-patterned field of a prepared PDMS stamp was cut out and washed by flushing with ethanol (100%) and distilled water. The stamp was then dried with nitrogen. 10 μl of BSA-Cy5-Biotin solution [20] were then pipetted onto the μ-patterned field and the stamp was incubated for 15 min in the dark at room temperature. Afterwards the stamp was washed with phosphate buffered saline (PBS) and distilled water and again dried with nitrogen. The stamp was then placed on a streptavidin-coated glass slide (Xenopore, Hawthorne, New Jersey) and incubated for 30 min in the dark at room temperature. After removal of the PDMS stamp a Secure Seal Hybridization Chamber (Sigma; Schnellendorf, Germany) was placed on the μ-contact-printed field. The reaction chamber was filled with 60 μl of biotinylated antibody solution (10 μg/ml in PBST) and incubated for 30 min in the dark at room temperature. The sample was then finally washed with PBST to remove excess antibodies and cells were grown for at least 3–4 hours to allow cell attachment.

### Live cell TIRF microscopy

The detection system was set up on an epi-fluorescence microscope (Olympus IX81). Diode lasers (Toptica Photonics, Munich, Germany) were used for selective fluorescence excitation of GFP, YFP and Cy5/DiD at 488 nm, 514 nm and 640 nm, respectively. A 405 nm diode laser (Toptica Photonics, Munich, Germany) was used for bleaching of GFP/YFP fluorescence. Samples were illuminated in total internal reflection (TIR) configuration (CellTIRF, Olympus) using a 60 x oil immersion objective (NA  =  1.49, APON 60XO TIRF, Olympus, Munich, Germany). After appropriate filtering using standard filter sets, fluorescence was imaged onto a CCD camera (Orca-R2, Hamamatsu, Japan). Samples were mounted on an x-y-stage (CMR-STG-MHIX2-motorized table; Märzhäuser, Germany) and scanning of larger areas was supported by a laser-guided automated focus-hold system (ZDC-2; Olympus). For FRAP experiments single patterns were photobleached with a laser pulse (405 nm) applied for 100 ms. Recovery images were recorded at indicated time intervals. FRAP images were analyzed using the Multimeasure plugin of ImageJ [27]. Data were normalized by the pre-bleach image and curve fitting was done using Graphpad Prism. Resulting FRAP curves were plotted based on the standard error of the mean (SEM) and fitted using a bi-exponential equation. Kinetic FRAP parameters were directly obtained from curve fitting.

### Data analysis

Initial imaging recordings were supported by the Olympus XcellenceRT software. Images were exported as TIRF-frames and fluorescence contrast analysis was performed using the Spotty framework [28]. Briefly, the BSA-Cy5 image was used for a self-adaptive algorithm to identify the grid and antibody coated regions. Based on the correct identification of the grid position with respect to fluorescent patterns, the fluorescence contrast C was calculated as C =  (F^+^–F^−^)/(F^+^–F_BG_), where F^+^ denotes the intensity of the inner pixels of the μ-pattern, F^−^ the intensity of the surrounding pixels of the pattern and F_BG_ the intensity of the global background as originally described [21]. Other calculations and fittings were done using Microsoft Excel and Graphpad Prism software.

### Cytotoxicity assay

Cytotoxic effects of various compounds used for EGFR inhibition were evaluated by using a resazurin-based *in-vitro* toxicology assay (Sigma; Schnellendorf, Germany) according to the instructions of the manufacturer. In short, HeLa cells were seeded in 96-well plates (40.000 cells/well) and grown overnight, followed by treatment with the indicated compound for 24 hours. Subsequently, the cells were incubated with a medium containing 10 % resazurin for 2 hours. The reduced form of resazurin (resorufin) was then determined by the use of a microplate reader in fluorescence mode (Polarstar, BMG Labtech; Ortenberg, Germany). Cell viability was normalized to non-treated cells grown under the same conditions.

### DiD stain

DiD oil was reconstituted according to manufacturer's instructions. Live cell membrane staining was done directly on the μ-biochip by pipetting 1 μl of reconstituted DiD oil into the incubation chamber followed by gentle mixing of the solution. Samples were incubated for 20–30 min at 37°C and washed twice with a medium prior to analysis.

## Results

### Setup of TIRF microscopy guided EGFR screening assay

**Figure 1:**
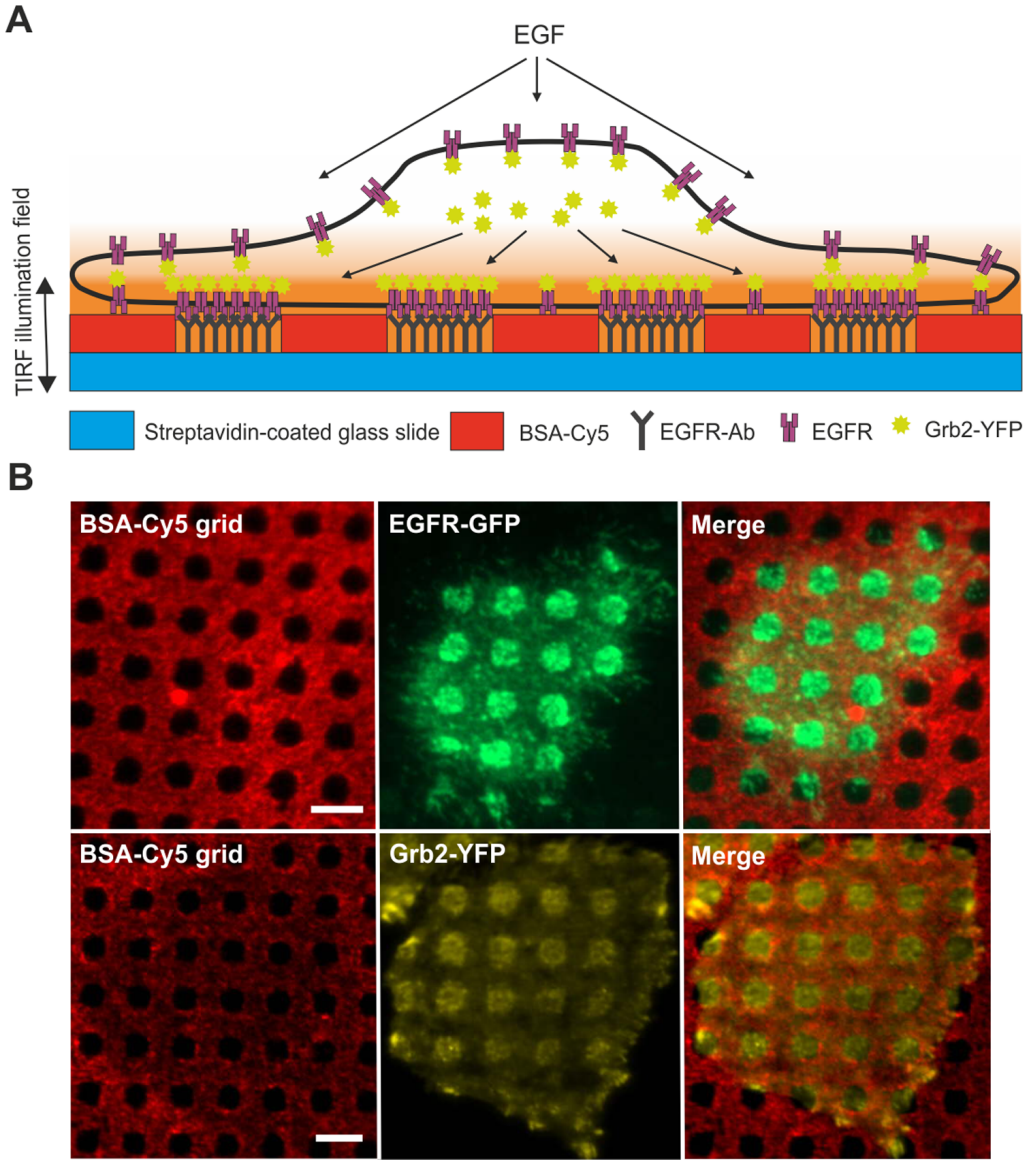
Basic principle of the μ-patterning screening assay. (A) Schematic diagram of EGFR-Grb2 interaction in living cells grown on a functionalized μ-biochip. Cells attach within 2–3 hours after seeding on the μstructured glass surface and endogenous EGFR is captured by anti-EGFR antibodies. Specific interaction of fluorescently labeled Grb2 with the EGFR is characterized by enhanced co-patterning in anti-EGFR antibody enriched regions. (B) Proof of principle. HeLa cells expressing EGFR-GFP are grown on an anti-EGFR antibody coated μ-biochip. Accurate alignment of anti-EGFR antibodies into unblocked spots leads to a strong enrichment of the bait protein (EGFR) into μ-patterns (upper row). Co-localization of the fluorescently labeled prey protein (Grb2-YFP) in anti-EGFR antibody positive regions indicates specific protein-protein interactions (lower row). Scale bars  =  5 μm.

We attached a capture antibody to the extracellular domain of the EGFR (bait) in a characteristic μ-pattern on the surface of a glass coverslip (Figure 1A). This experimental setup should lead to the redistribution of EGFR interacting proteins (bait) within the same μ-patterns on the plasma membrane. As a prey molecule, we chose the EGFR interacting adaptor molecule Grb2 [29] labeled with YFP [30]. Specific interaction should result in the co-enrichment in EGFR-concentrated areas. For our experiments we used the HeLa cell line, which has been reported to express high levels of endogenous EGFR and is therefore frequently used in cancer research [31]–[33]. Furthermore, as a prerequisite for the μ-patterning assay, HeLa cells were shown to have a flat interface with the μ-biochip as confirmed by staining with the lipophilic tracer DiD (Figure S1). We could therefor exclude false-positive signals because of tighter contact of the patterned spots with the coverslip, which could lead to better illumination in the evanescent field.

### Inducibility and quantification of EGFR-Grb2 interaction

**Figure 2:**
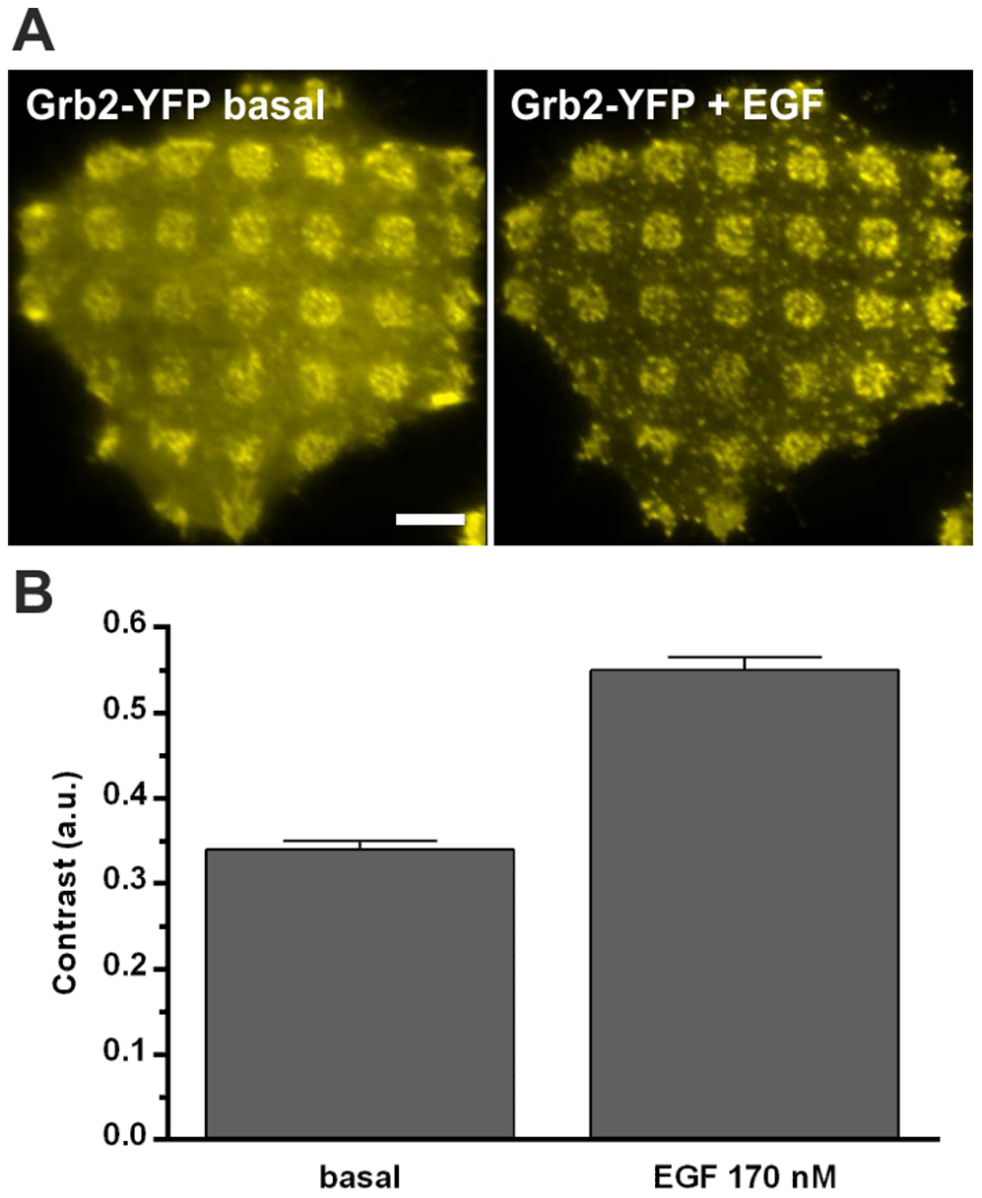
Inducibility of EGFR signaling on μ-structured surfaces. (A) Representative HeLa cell expressing Grb2-YFP grown on an anti-EGFR antibody coated μ-biochip. Induction by EGF (170 nM) for 15 minutes significantly increases the basic fluorescent contrast. (B) Quantification of EGFR-Grb2 interaction by contrast evaluation. Addition of EGF leads to an increase in the Grb2 contrast from ∼0.33 to ∼0.55. Error bars are based on the standard error of the mean (n > 300 cells). Scale bar  =  5 μm.

In a first attempt we analyzed the redistribution of the EGFR in HeLa cells grown on anti-EGFR antibody coated μ-biochips. Therefore, we generated a clone stably expressing GFP-labeled EGFR. As shown in Figure 1B (upper row) EGFR-GFP enrichment correlated well with anti-EGFR antibody positive regions. Next, we studied the interaction between endogenous EGFR and fluorescent Grb2. These experiments were performed in HeLa cells stably expressing Grb2-YFP. We found a significant enrichment of bait Grb2 in EGFR-positive regions of the μ-patterns confirming the interaction of these two proteins (Figure 1B, lower row). This basic interaction between Grb2 and EGFR appeared without any additional EGFR activation and may be caused by the stimulating nature of the antibody coated surface and/or the presence of growth factor containing serum. Our experimental setup does not allow for serum depletion, since this leads to deficient cell attachment. However, the interaction of EGFR and Grb2 can be clearly stimulated; the addition of EGF resulted in a prominent increase of the Grb2-YFP fluorescence within EGFR-enriched μ-patterns. In addition, we also detected significant clustering of Grb2 indicating EGFR desensitization events in clathrin-coated pits (CCPs) (Figure 2A). For quantification of this remarkable difference we used an algorithm that allows for semiautomatic analysis of μ-patterns by calculating the fluorescence contrast <c> (Figure 2B), as performed in previous studies [21], [25]. Analysis of >300 cells resulted in a basal mean contrast <c> of 0.34±0.01 compared to 0.55±0.01 after EGF treatment for 15 min.

### Sensitivity of EGFR-Grb2 interaction

**Figure 3:**
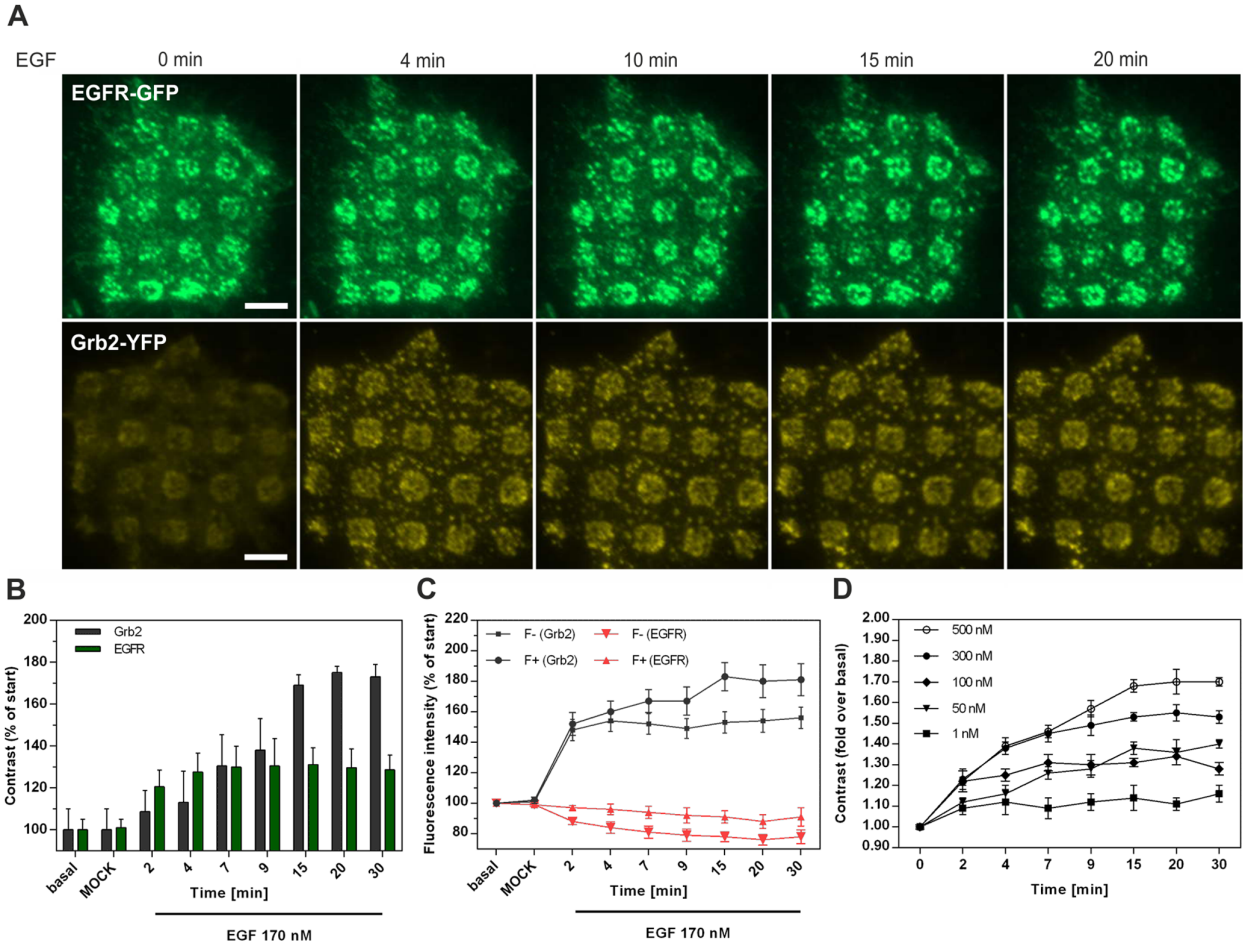
Sensitivity of EGFR-Grb2 interaction on μ-biochips. (A) HeLa cells expressing EGFR-GFP or Grb2-YFP, respectively, grown on anti-EGFR antibody coated μ-biochips at indicated time points after EGF (170 nM) addition. (B) Temporal resolution of the change in contrast induced by EGF (n = 40 cells). (C) Time course of the corresponding F+ and F- signals of EGFR and Grb2, respectively (n = 40 cells). (D) EGF concentration dependent increase of Grb2 contrast (n = 40). Fluorescent contrast was normalized to the value prior to EGF stimulation. Error bars are based on the standard error of the mean. Scale bar  = 5 μm.

Being aware that the increased Grb2 contrast might only be due an elevated enrichment of additional EGF receptors upon stimulation, we compared the fluorescence contrast of EGFR and Grb2 in a time dependent manner. Figure 3A shows representative EGFR-GFP (upper row) and Grb2-YFP (lower row) expressing cells grown on anti-EGFR antibody μ-biochips before and after EGF stimulation at given time points. Quantification of the respective fluorescence signal indicated that the Grb2 contrast increases to a larger extent than the one of the EGFR (Figure 3B). It exhibits a continuing rise after EGF stimulation and reached a plateau value after approximately 30 min (∼70% increase compared to the initial value), whereas the EGFR contrast leveled off after 4 min (∼30% increase compared to initial value). The increase in EGFR contrast upon stimulation is mainly caused by the loss of fluorescence intensity in regions outside the μ-patterns (F^−^), which can be explained by immediate receptor internalization processes [30], [34], [35] that preferentially occur in the absence of anti-EGFR antibodies (Figure 3C, red time course). Contrarily, the Grb2 contrast increase is characterized by a steady rise of the fluorescence intensity in EGFR-enriched regions (F^+^), whereas the F^−^ signal remains stable after an initial increase (Figure 3C, black time course). To further investigate the sensitivity of our experimental system we quantified the Grb2 contrast at different EGF concentrations ranging from 1 to 500 nM (Figure 3D). While the lowest EGF concentration only led to a minimal response within the first two minutes after stimulation, a maximum effect was achieved with 500 nM EGF (1.7 fold contrast increase compared to start value).

### Characterization of different EGFR modulators

**Figure 4:**
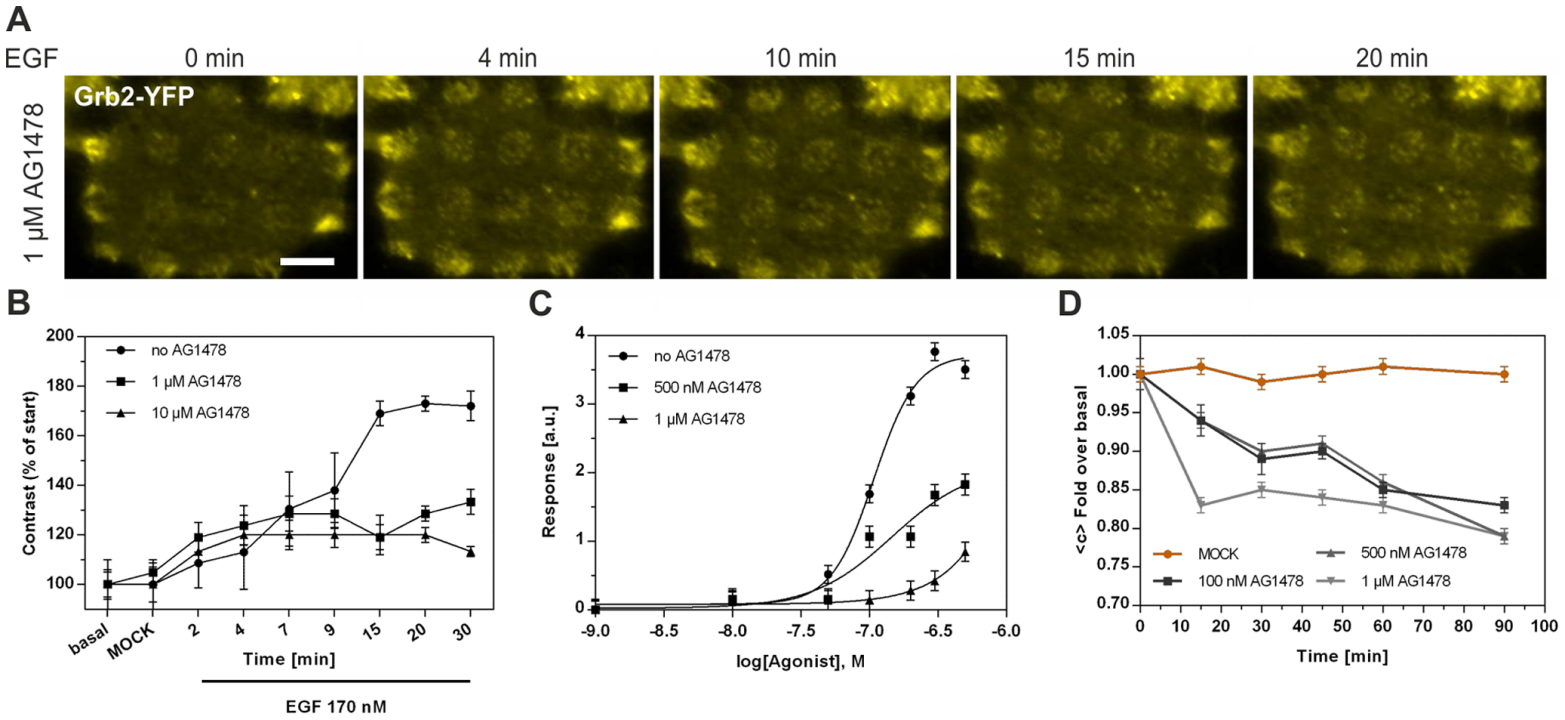
Inhibition of EGFR-Grb2 interaction by the small tyrosine kinase inhibitor AG1478. (A) Representative HeLa cell pretreated with 1 μM AG1478 for 4 hours expressing Grb2-YFP grown on an anti-EGFR antibody coated μ-biochip at the indicated time points after EGF addition (170 nM). (B) Time course of the Grb2 contrast change upon EGF stimulation in control and AG1478 pretreated (1, 10 μM) cells (n = 50). Pretreatment with a tyrosine kinase inhibitor leads to a decreased contrast compared to untreated cells. Fluorescent contrast was normalized to the value prior to EGF stimulation. (C) Dose-response relationship of the EGFR-Grb2 interaction in cells pretreated with indicated AG1478 concentrations (n = 40). (D) Temporal resolution of Grb2 contrast at different AG1478 concentrations. Cells were incubated with AG1478 and the decrease in contrast was observed for 90 min. Contrast was normalized to the value prior AG1478 addition (n =  40). Error bars are based on the standard error of the mean.

**Figure 5:**
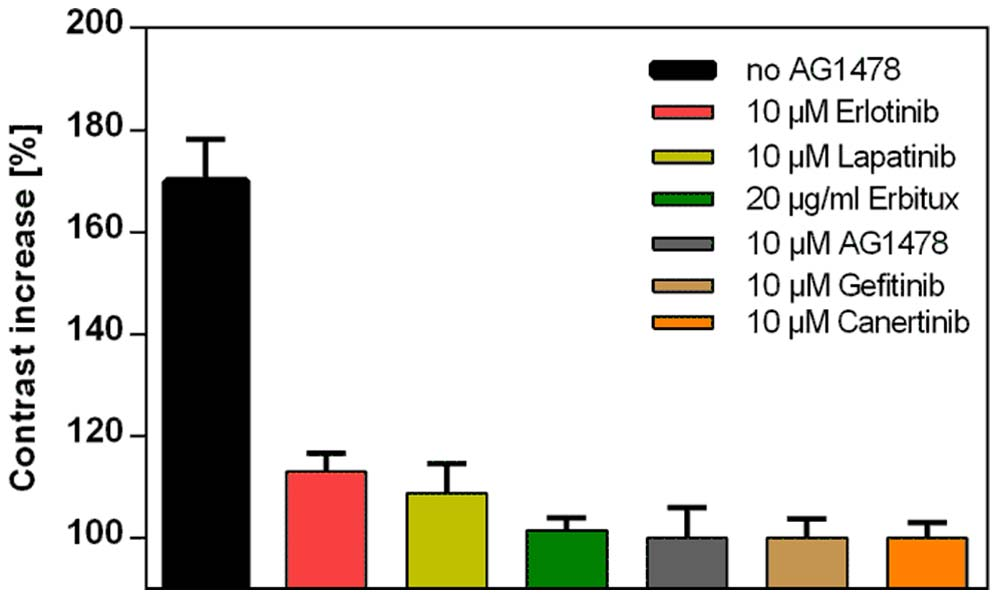
Inhibition of Grb2 recruitment to the EGFR by EGFR-activity modulating drugs. Grb2 contrast increase upon EGF stimulation (170 nM, 15 min) in cells pre-incubated with the indicated EGFR antagonists for 4 hours in comparison to untreated cells. Error bars are based on the standard error of the mean (n = 40 cells).

We continued our study by characterizing the effects of various EGFR inhibitors on the Grb2 contrast. First, cells pretreated with 1 μM AG1478 for 3 hours were grown on an anti-EGFR antibody coated μ-biochip. As shown in Figure 4A this procedure resulted in a slightly reduced basal and a significantly inhibited Grb2 contrast 20 min after EGF stimulation, respectively (Figure 4A and B). In addition, Grb2 clustering indicating the formation of CCPs was found to be significantly reduced: The increase of the fluorescence intensity of Grb2 in individual clusters was inhibited by ∼35% (Figure S2). Next, we pretreated the cells with different AG1478 concentrations (0.5 and 1 μM) and analyzed the Grb2 contrast at increasing EGF concentrations. As shown in Figure 4C, we were able to set up a dose-response relationship (EC50 no AG1478  =  107 nM, EC50 500 nM AG1478  =  146 nM, EC50 1 μM AG1478  =  303 nM), a prerequisite for in-depth characterization of medically relevant substances. Furthermore, time-resolved analysis proved an AG1478 concentration dependent decrease of the Grb2-contrast (Figure 4D). We conclude that quantification of the Grb2 contrast increase after EGF stimulation in cells pretreated with an EGFR inhibitor is a convenient way to determine the efficacy of those substances. Thus, this EGFR inducibility in terms of Grb2 contrast development was investigated in cells pretreated with further, clinically tested EGFR antagonistic molecules. As expected, Grb2 recruitment was significantly down-regulated in cells incubated with the small kinase inhibitors Canertinib, Erlotinib, Lapatinib, Gefitinib and the monoclonal antibody Erbitux (Figure 5): We found between 90% and almost complete inhibition of Grb2 contrast increase upon stimulation by 170 nM EGF. Gefitinib and Canertinib appeared to be the most potent inhibitors of the tested drugs. Importantly, cells remained fully viable at the chosen antagonist concentration (Figure S3).

### The nature of CCP formation on μ-biochips

**Figure 6:**
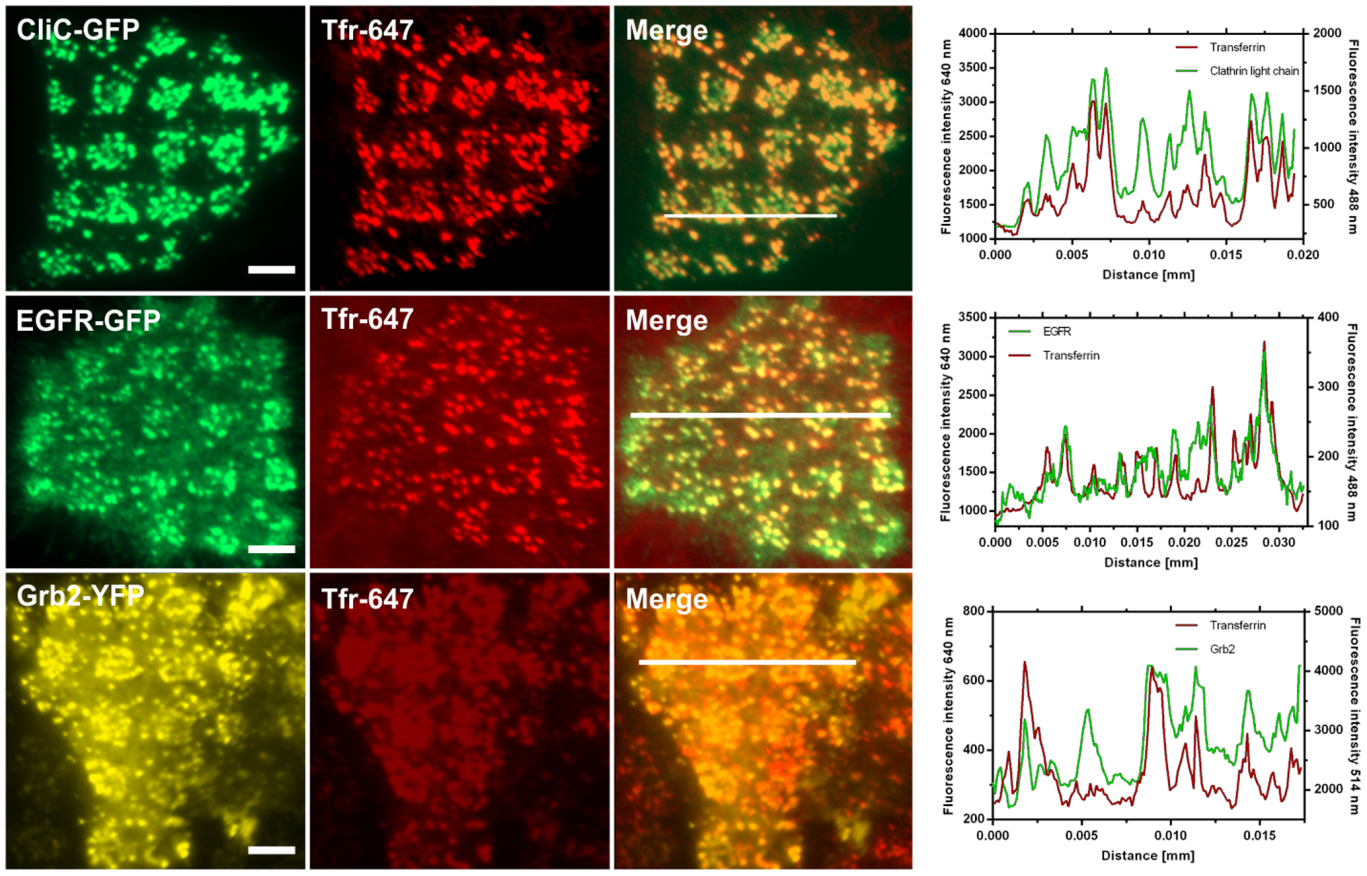
Accumulation of EGFR and Grb2 in clathrin-coated pits (CCPs). Representative TIRF microscopy images of HeLa cells transfected with clathrin light chain-GFP (CliC-GFP), EGFR-GFP or Grb2-YFP, respectively, co-stained by Transferrin-Alexa647 as a marker for CCPs. Cells were stimulated with EGF for 15 min with simultaneous addition of Tfr-647 (25 μg/ml). Fluorescence intensity profiles are shown on the right for the indicated line scans. Scale bars  = 5 μm.

**Figure 7:**
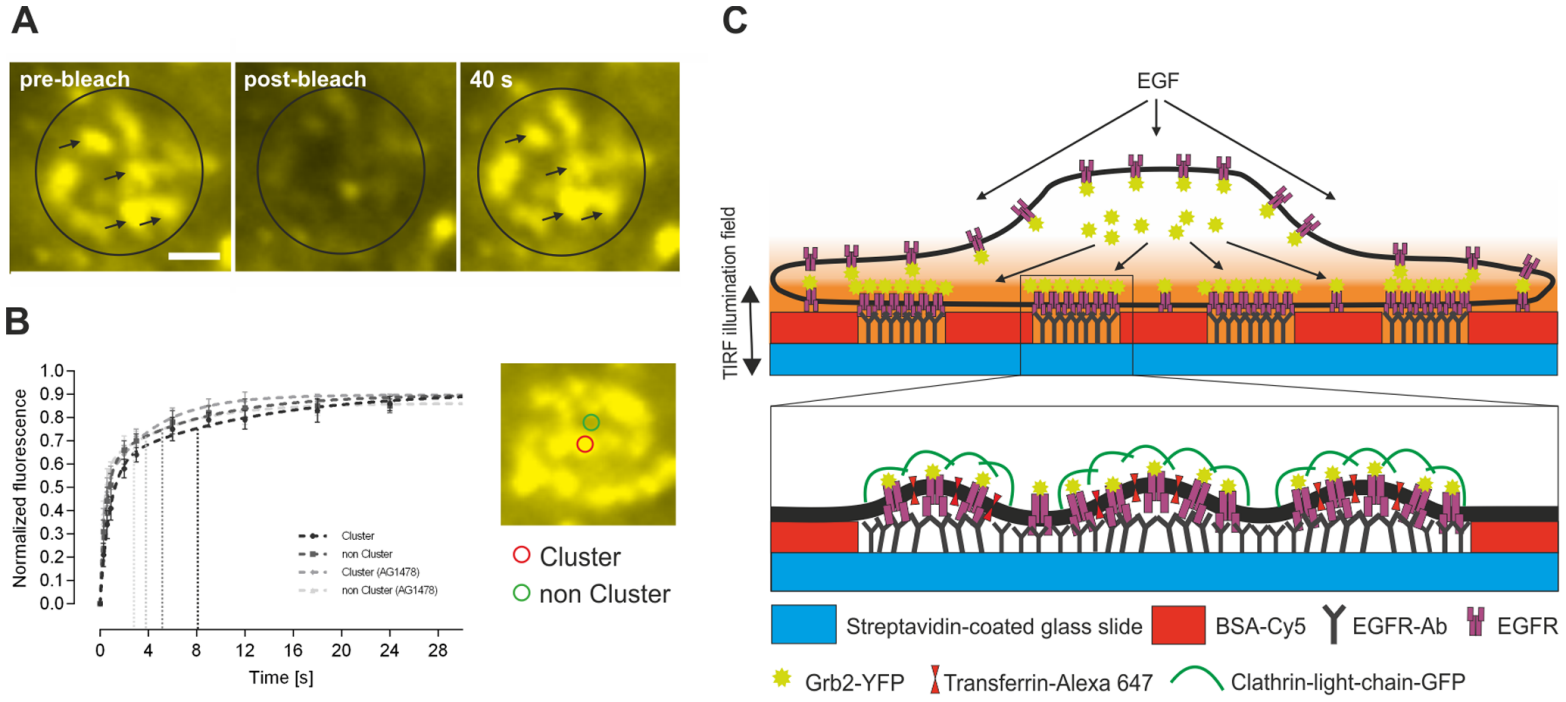
Stabilization of CCPs on the μ-patterned surface. (A) Representative FRAP images of a single pattern in HeLa cells expressing Grb2-YFP after EGF stimulation. Preformed CCPs remain stable and are not endocytosed as indicated by recovery of Grb2 at the same position (black arrows). (B) Mean fluorescence recovery curves of bleached cluster (red circle)/non-cluster (green circle) regions within a single pattern of untreated/AG1478-treated (1 μM for 4 hours) cells. Vertical dashed lines represent slow half-times of the different samples. Error bars are based on the standard error of the mean (n = 40 for cluster/non-cluster regions of 10 different cells). Scale bar  =  1 μm. (C) Cartoon indicating the formation and stabilization of CCPs on anti-EGFR antibody coated μ-biochips.

EGF binding leads to a rapid internalization of activated receptors into lysosomes for degradation [36]. CCPs represent the main structures for the endocytosis of activated EGFRs [30], and the interaction of Grb2 with the EGFR plays a decisive role in the initial steps of clathrin-mediated internalization [30], [37]. Based on the observed clustering of Grb2, preferentially in EGFR enriched regions, we investigated the nature of CCP formation on the μ-biochip. First, using 2-color TIRF microscopy we confirmed that CCPs are effectively abundant in anti-EGFR antibody positive regions. For this purpose we grew HeLa cells expressing a clathrin light chain fused to GFP (Clic-GFP) on the EGFR μ-biochip and co-stained the cells with Alexa-647 conjugated transferrin (Tfr-647), a standard marker for CCPs [38], [39] (Figure 6, upper row). Next, we determined the degree of co-localization of EGFR-GFP and Grb2-YFP, respectively, with Tfr-647. The obtained results indicate a significant concentration of both EGFR and Grb2 in CCPs (Figure 6, middle and lower row). This co-localization was evident by line scan analysis (Pearson’s coefficient CliC-Tfr  = 0.85; EGFR-Tfr  = 0.75; Grb2-Tfr  = 0.76). We continued our work by analyzing the relevance of CCPs for the binding of Grb2 to the EGFR. As shown in a previous study, the μ-patterning assay is a suitable tool to study interaction kinetics in a live cell context [21]. Using the fluorescence recovery after photobleaching (FRAP) technique we bleached single patterns on the μ-biochip and determined the recovery of the Grb2 fluorescence. As shown in Figure 7A, Grb2 was found to be highly mobile resulting in a 90% recovery within 40 seconds for EGF-stimulated cells. Importantly, the recovery appeared in the same clusters, which clearly shows that the generated CCPs remain stable within the μ-patterns. The CCPs were found to be stable in EGFR positive areas for up to 60 min, while their stability was significantly reduced outside the μ-patterns (Figure S4). We speculate that the fixation of a high number of EGFR molecules on the μ-biochip stabilizes CCPs in an early stage and prevents their internalization (Figure 7C). In any case, our experiments clearly show that there is an active exchange of Grb2 molecules at the EGFR enriched in CCPs. Finally, we quantified the increase in Grb2 fluorescence upon EGF stimulation inside and outside the assembled CCPs of single μ-patterns. Therefore, we were able to calculate the recovery-rates and mobile fractions of individual clusters (encircled in red) and CCP-free interspaces (marked in green), respectively. While the mobile fractions appeared identical in both cases, we detected significantly decreased exchange-rates for Grb2 in CCPs. Pretreatment of the cells with AG1478 further increased these exchange-rates (Figure 7B and Table 1). Those results fit the model of an increased retention time of Grb2 bound to the activated EGFR.

**Table 1:** Kinetic FRAP parameters. Results are given as mean ± SEM.

	Cluster	non Cluster	Cluster (AG1478)	non Cluster (AG1478)
Plateau	0.91±0.01	0.90±0.01	0.89±0.01	0.86±0.01
K_Fast_	1.22±0.11	1.99±0.22	3.67±0.61	3.19±0.36
K_Slow_	0.084±0.016	0.131±0.027	0.241±0.036	0.177±0.033
Fast Half-Life	0.57±0.05	0.35±0.04	0.19±0.03	0.22±0.02
Slow Half-Life	8.30±1.57	5.29±1.15	2.88±0.43	3.91±0.74

40 cluster/non-cluster regions of 10 different cells were analyzed.

## Discussion

The epidermal growth factor receptor (EGFR) is one of the most extensively studied tyrosine kinase receptors, especially due to its role for the development of several solid tumors [40]. EGFR overactivity may lead to sustained signals for anti-apoptosis, cell proliferation, angiogenesis and metastasis, the basic properties of cancer [41]. Activated EGFR mainly acts via the Ras and Raf/MEK/ERK downstream pathway where the adaptor protein Grb2 is the key player through direct (Y1068 and Y1086) or indirect binding (via Shc) of the autophosphorylated receptor, respectively [26], [42]. Within recent years, several substances have been identified and clinically tested which inhibit EGFR activation. Those include monoclonal antibodies (mAb) directed towards the extracellular domain of the EGFR (e.g. Erbitux) and small molecule tyrosine kinase inhibitors (TKIs) that interfere with intracellular receptor signaling by inhibiting the catalytic kinase domain (e.g. Erlotinib, Canertinib, Gefitinib or Lapatinib). However, due to evolved resistances and insufficient efficacy there is an urgent need for further EGFR-activity inhibiting drugs. Importantly, this involves new test systems for the identification and better characterization of candidate substances, ideally in living cells. In this study we describe the usability of the μ-patterning assay [20] to quantify the inhibitory effects of various lead substances. Our method does not solely determine the phosphorylation status of the EGFR, but instead quantifies the recruitment of the adaptor protein Grb2 to the EGFR as a consequence of receptor activation. Thus, it represents a functional approach to the quantification of the activity of a key player in EGFR mediated signaling.

In comparison to other methods our strategy offers several advantages: First, the measurements do not depend on cell lysis and protein extraction steps and thus can be performed in living cells. Secondly, the experimental setup includes stable expression of bait (endogenous) and prey (ectopic expression), utilization of a TIRF microscopy setup with an automated focus-hold system and a high-precision motorized scanning table enabling image recording of hundreds of cells within a few minutes, and optimized algorithms for the quantitative extraction of relevant data [28]. In addition, current development in our lab aims to generate a multi-well imaging plate (96- and 384-well design) with a pre-functionalized and μ-structured surface of optimized configuration (e.g. smaller feature size or lines instead of patterns) and aseptic packaging. In combination with easily operable, microplate-readable TIRF scanners [43] high throughput rates for the screening of libraries appear achievable. Thirdly, the targeted signaling events can be stabilized. We were able to confirm the enrichment of the EGFR-Grb2 complex in CCPs. Interestingly, the interaction of the EGFR with the bait antibody stabilizes forming CCPs at the position of the μ-patterns, and thus prevents internalization of the receptor. This stabilization enables the characterization of signaling events on the very spot before/after drug addition over a long time period, even in the same CCPs. The EGFR is likely to remain its activity in these stabilized CCPs, consistent with a reported sustained Grb2 exchange also in endocytosed EGFR containing vesicles [44]. We are aware that the *in-vitro* system presented herein might not fully reflect the physiological conditions. However, as it can be seen by the change in contrast, addition of EGF clearly stimulates the binding of Grb2 to the receptor. In addition, antibody immobilization does not inhibit the formation of EGFR clustering within the μ-patterns, indicating an active signaling system. Fourthly, specific signals can be quantified independent from intracellular fluorescence. Application of TIRF microscopy allows for specific excitation of Grb2 molecules near the cell membrane and therefore minimizes unspecific fluorescence signals from the cytosol. Finally, the assay provides additional biological insight: for the present study we used the μ-patterning approach for a quantitative analysis of the EGFR-Grb2 interaction. It allows for a characterization of EGFR modulating drugs. However, our experimental setup provides additional information on the molecular mechanisms of bait-prey interaction: here we were able to estimate the interaction strength (fluorescent contrast) and calculate the residence time of Grb2 at the EGFR by determining the exchange rate. Our results indicate a fast exchange of Grb2 molecules, which is significantly decreased upon receptor activation and increased upon receptor inhibition by AG1478. To our knowledge, there is only a single further study performed on live cells that reports on the binding kinetics of these two molecules [45]. Data obtained from these single molecule microscopy experiments are in good agreement with the exchange rates identified in our study. A previous study reported on multiple-state reactions between the EGFR and Grb2, and the binding kinetics were explained using a multiple-exponential function [46]. Here we used a bi-exponential fit (best fit results) to characterize the exchange kinetics of Grb2 in CCPs. We speculate that the fast components represent cytosolic diffusion of Grb2 and the slow components further describe the binding reactions between the EGFR and Grb2 in CCPs. However, our results clearly indicate an ongoing exchange of Grb2 with the EGFR localized to CCPs.

One question that might need additional attention in the near future is the apparent pre-activation of the EGFR on our μ-biochip. Unfortunately, pre-starvation prevents the cells from proper adhesion to the surface. Furthermore, the stimulating potential of the anti-EGFR antibody may induce phosphorylation events due to receptor dimerization, and finally lead to a significant contrast already in unstimulated cells. While the stimulating potential of EGF in our system (i.e. it almost doubles the fluorescent Grb2 contrast) is unquestioned and in principle sufficient for the characterization of EGFR activity modulating substances, we aim to reduce this limitation. For this reason, fragment antigen-binding (Fabs) molecules may be used instead of full antibodies. Another possibility would be the application of single-domain antibodies, which are even smaller than Fabs, and already exist as an EGFR selective version and do not show any intrinsic activity [47]. Both full antibody alternatives offer the advantage of being monovalent and highly selective binding molecules. However, these molecules are much more expensive than conventional antibodies that can only be obtained in biotinylated form at low costs.

## Supporting Information

Figure S1 DiD membrane stain. HeLa cells expressing Grb2-YFP were grown on an anti-EGFR antibody coated μ-biochip for 4 hours and incubated with EGF (170 nM) for 15 min. Cell membrane was then uniformly labeled by the lipophilic tracer DiD confirming sufficient attachment of the cells to the functionalized surface. Scale bar  =  3 μm.
(TIF)

Figure S2 Analysis of fluorescence increase of single clusters. HeLa cells were pretreated with 1 μM AG1478 for 4 hours and the fluorescent signal of Grb2-YFP containing clusters after stimulation by EGF (170 nM for 15 min) was compared to non-treated control cells.
(TIF)

Figure S3 Cytotoxicity of EGFR inhibitors. HeLa cells were grown in 96-well plates (50,000 cells/well) over night. Inhibitors were added to the cells at indicated concentrations for 24 hours. Cytotoxicity was measured using a resazurin-based in-vitro toxicology assay.
(TIF)

Figure S4 Stabilization of CCPs on the **μ**-biochip. HeLa cells expressing Grb2-YFP were stimulated with EGF (170 nM) and cluster formation was observed for 50 min. Clusters within the bait-enriched region (purple arrows) remained stable for up to 50 min, whereas clusters in the bait-free area (yellow arrows) disappeared due to endocytotic events. Scale bar  =  3 μm.
(TIF)
