# Supplementary figures and images for: Quantification and Kinetic Analysis of Grb2-EGFR Interaction on Micro-Patterned Surfaces for the Characterization of EGFR-Modulating Substances
Source: PLoS One. 2014 Mar 21;9(3):e92151. doi: 10.1371/journal.pone.0092151 (PMC3962377; doi:10.1371/journal.pone.0092151)

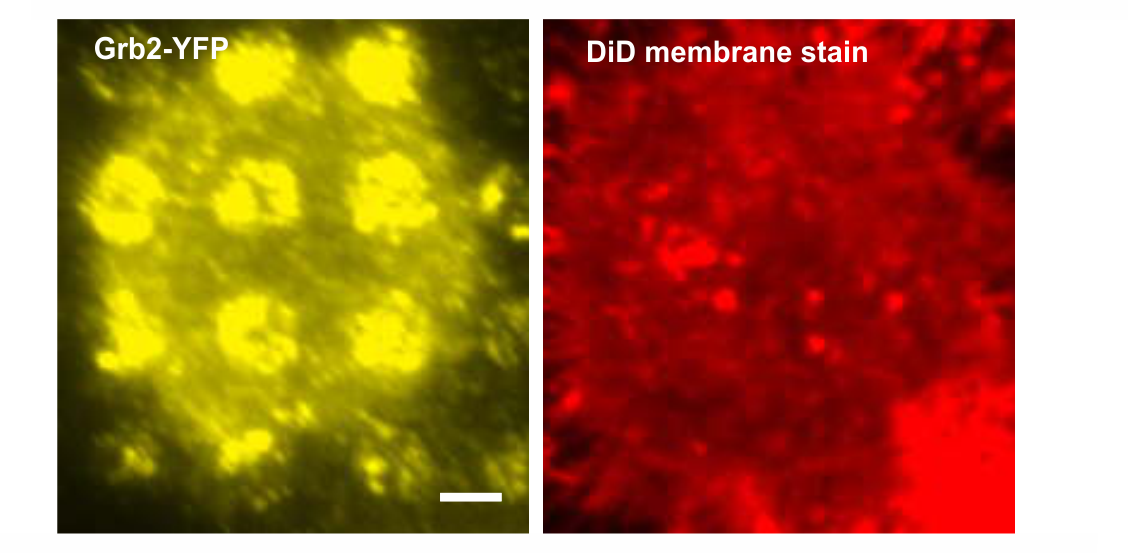

Supplement: Figure S1 — DiD membrane stain. HeLa cells expressing Grb2-YFP were grown on an anti-EGFR antibody coated μ-biochip for 4 hours and incubated with EGF (170 nM) for 15 min. Cell membrane was then uniformly labeled by the lipophilic tracer DiD confirming sufficient attachment of the cells to the functionalized surface. Scale bar = 3 μm. (TIF) [file pone.0092151.s001.tif]

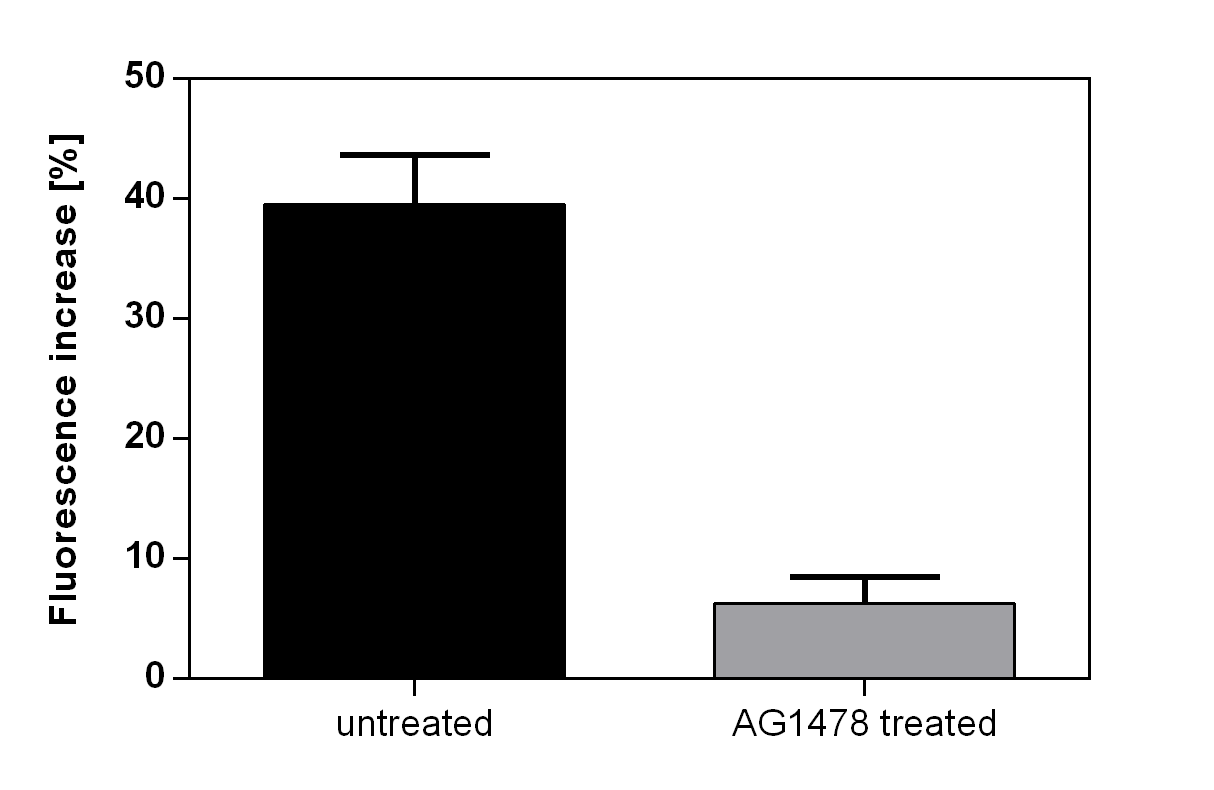

Supplement: Figure S2 — Analysis of fluorescence increase of single clusters. HeLa cells were pretreated with 1 μM AG1478 for 4 hours and the fluorescent signal of Grb2-YFP containing clusters after stimulation by EGF (170 nM for 15 min) was compared to non-treated control cells. (TIF) [file pone.0092151.s002.tif]

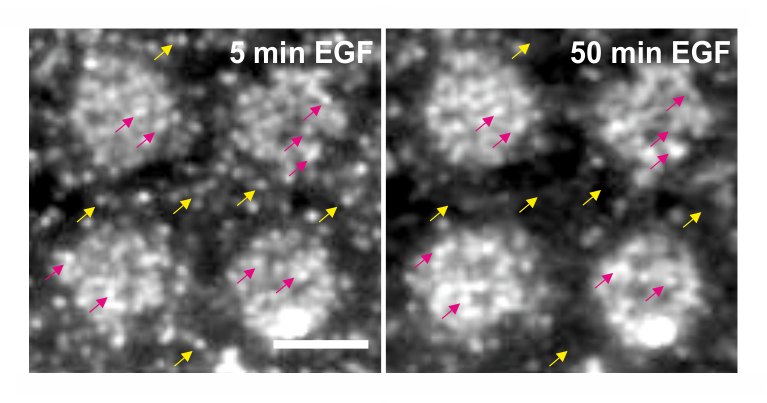

Supplement: Figure S4 — Stabilization of CCPs on the μ-biochip. HeLa cells expressing Grb2-YFP were stimulated with EGF (170 nM) and cluster formation was observed for 50 min. Clusters within the bait-enriched region (purple arrows) remained stable for up to 50 min, whereas clusters in the bait-free area (yellow arrows) disappeared due to endocytotic events. Scale bar = 3 μm. (TIF) [file pone.0092151.s004.tif]
